# The growing problem of obesity in South Africa

**DOI:** 10.4102/safp.v67i1.6001

**Published:** 2025-01-01

**Authors:** Indiran Govender, Alethea Sunnasy

**Affiliations:** 1 Department Family Medicine and Primary Health Care, Faculty of Health Sciences, Sefako Makgatho Healthcare Sciences University, Pretoria, South Africa; 2 Department of Nursing, Faculty of Health Sciences, University of Johannesburg, Johannesburg, South Africa

## Introduction

The World Health Organization (WHO) reports that obesity is one of the major problems that leads to a range of health complications, which puts a strain on the already strained healthcare systems.^1^ It was estimated that 39% of adults are overweight while 13% are obese globally, and these numbers are increasing.^1^ In South Africa (SA), there seems to be little action taken by adults, children, healthcare workers and politicians to address the serious problem of obesity, which is glaring in our public and private healthcare systems, and which presents with disastrous health management challenges.^2^

The term obesity may be described differently by different authorities; the layperson may define it as unsightly or unwanted fat on the body.^1^ The WHO defines obesity as a chronic and intricate condition marked by the accumulation of excess body fat.^3^ According to the Global Obesity Observatory, SA overweight refers to a body mass index (BMI) between 25 kg/m^2^ and 29.9 kg/m^2^, and obesity refers to a BMI greater than 30 kg/m^2^. The BMI serves as a recognised method for categorising the extent of overweight or obesity in an individual.^4^ Having too much body fat, especially around the waistline line increases the risk of developing health-related problems, for women, a high-risk waistline is defined as 80 cm or greater, while for men, it is 94 cm or more.^5^ Obesity commonly occurs when a person’s energy intake exceeds their energy expenditure over a sustained period. Key factors that influence this energy balance include the type of food consumed and physical activity.^2^ Economic transition has contributed significantly to obesity by having living environments situated close to places that sell unhealthy food and beverages.^2^ The obesity epidemic emerges from a complex interplay of environmental factors, genetic predisposition and human behaviour, forming a multifaceted and intricate dynamic.^6^ There is an increased intake of energy-dense foods, which are high in fat coupled with an increase in physical inactivity owing to the increasingly sedentary nature of many forms of work, changing modes of transportation and increasing urbanisation.^2^

Since 1990, the rate of obesity among adults worldwide has more than doubled, while adolescent obesity has quadrupled.^4^ In 2022, the incidence of obesity globally stood at one in eight individuals. While the degree of awareness varies among countries, the general lack of public acknowledgement regarding obesity and its negative effects poses a significant obstacle to effectively addressing and managing this issue. Certain cultures tend to favour larger body sizes and exhibit less tolerance for thinness, particularly in women.^7^ Additionally, there is a global preference for heavier children.^7^ One would expect obesity to be a problem only in rich first-world nations; however, recent data suggest a rapid increase in obesity rates within low- and middle-income countries.^7^

In SA, obesity is far more prevalent among women than men, with the prevalence of obesity among women of reproductive age increasing from 51.3% to 60% between 1988 and 2017.^8^ Approximately 31% of men and 68% of women are classified as obese.^5^ In SA, obesity among women typically begins at a young age, with 10% of women aged 15–24 already classified as obese. Obesity is higher among black females (about a third are obese). This underscores the importance of initiating primary prevention efforts early, especially for girls. Among African women, urban residents exhibit the highest obesity rates. Interestingly, education level influences BMI, with women lacking formal education having lower BMIs, possibly because of more manual labour. Women with tertiary education also have lower BMIs, possibly influenced by health awareness or societal beauty standards. Obesity management is particularly crucial for older women across all demographic groups.^8^ Among men, the urbanised, higher-educated and older white men have elevated obesity rates.^8^

## Why the concern with obesity?

Obesity leads to a myriad of health problems. Some of these common non-communicable diseases include type 2 diabetes mellitus, hypertension, cardiovascular diseases, metabolic syndrome, as well as many forms of cancer.^9^ Ensuring the prevention of childhood obesity is crucial for averting complications that may arise in adolescence, early adulthood and later stages of life. Obesity in children is recognised as a multifaceted condition with potentially adverse consequences and complications, such as hyperinsulinaemia and insulin resistance in children under the age of five. Additionally, it may lead to hyperandrogenism, polycystic ovarian syndrome, hypertension, dyslipidaemia, chronic inflammation, increased tendency for blood clotting, endothelial dysfunction, as well as asthma, gastrointestinal issues and neurological disorders.^9^

The common health consequences of obesity include strokes, musculoskeletal disorders (such as osteoarthritis), and cancer, specifically endometrial, breast and colonic, gall bladder disease, gallstones, gout and breathing problems, such as sleep apnoea, are other consequences of obesity, which reduce life expectancy. There is also a higher prevalence of mental illness, such as depression, anxiety and other mental disorders.^10^

## Conclusion

Obesity represents a significant health challenge, demanding increased attention to its ramifications as a non-communicable disease in both adults and children. These may be referred to diseases of affluence. Formal education seems to be protective, while black women and white men are at an increased risk. Governmental action is necessary to decrease the fat and sugar levels in food products. Implementing exercise initiatives and interventions within schools is vital for combating childhood obesity. Parents and healthcare workers need to continuously advocate for healthy lifestyles and weight control. Engaging in community advocacy for healthy lifestyles from an early age, with heightened parental participation, is essential.

In the United Kingdom (UK), the National Health System currently spends £47 billion a year (approximately R982 billion a year) dealing with the healthcare and social costs of an increasingly overweight population. The cost is said to be a greater burden on the UK’s economy than armed violence, war and terrorism. The SA does not have the resources to support spending on obesity, for example on bariatric surgery, equipment, larger and wider beds and wider-access doors in hospitals for obese patients. The evidence suggests that the economic and societal impact of obesity is deep and lasting.
